# Transdermal Influenza Immunization with Vaccine-Coated Microneedle Arrays

**DOI:** 10.1371/journal.pone.0004773

**Published:** 2009-03-10

**Authors:** Dimitrios G. Koutsonanos, Maria del Pilar Martin, Vladimir G. Zarnitsyn, Sean P. Sullivan, Richard W. Compans, Mark R. Prausnitz, Ioanna Skountzou

**Affiliations:** 1 Department of Microbiology & Immunology and Emory Vaccine Center, Emory University School of Medicine, Atlanta, Georgia, United States of America; 2 School of Chemical and Biomolecular Engineering, Georgia Institute of Technology, Atlanta, Georgia, United States of America; New York University School of Medicine, United States of America

## Abstract

**Background:**

Influenza is a contagious disease caused by a pathogenic virus, with outbreaks all over the world and thousands of hospitalizations and deaths every year. Due to virus antigenic drift and short-lived immune responses, annual vaccination is required. However, vaccine coverage is incomplete, and improvement in immunization is needed. The objective of this study is to investigate a novel method for transdermal delivery using metal microneedle arrays (MN) coated with inactivated influenza virus to determine whether this route is a simpler and safer approach than the conventional immunization, capable to induce robust immune responses and confer protection against lethal virus challenge.

**Methodology/Principal Findings:**

Inactivated A/Aichi/2/68 (H3N2) influenza virus was coated on metal microneedle arrays and applied to mice as a vaccine in the caudal dorsal skin area. Substantial antibody titers with hemagglutination inhibition activity were detected in sera collected two and four weeks after a single vaccine dose. Challenge studies in mice with 5×LD_50_ of mouse adapted Aichi virus demonstrated complete protection. Microneedle vaccination induced a broad spectrum of immune responses including CD4+ and CD8+ responses in the spleen and draining lymph node, a high frequency of antigen-secreting cells in the lung and induction of virus-specific memory B-cells. In addition, the use of MN showed a dose-sparing effect and a strong Th2 bias when compared to an intramuscular (IM) reference immunization.

**Conclusions/Significance:**

The present results show that delivery of inactivated influenza virus through the skin using metal microneedle arrays induced strong humoral and cellular immune responses capable of conferring protection against virus challenge as efficiently as intramuscular immunization, which is the standard vaccination route. In view of the convenience of delivery and the potential for self-administration, vaccine-coated metal microneedles may provide a novel and highly effective immunization method.

## Introduction

Transdermal delivery of bioactive compounds on the skin has been used for thousands of years for therapeutic or prophylactic purposes, and more than one billion patches are now sold annually for delivery of small molecule drugs [1]. While the skin permits entry of certain low molecular weight compounds at therapeutic rates, the skin's barrier layer of stratum corneum generally blocks penetration of macromolecules and vaccines. Vaccine delivery to the skin therefore typically requires the use of a needle; either a hollow hypodermic needle or a solid bifurcated needle as used for scarification to administer smallpox vaccine [2], [3], [4].

Vaccine delivery to the skin is attractive because of the complex immunologic network that the skin provides [5]. The skin is extremely dense in innate immune cell populations whose role is to recognize, uptake and present foreign antigens to T and B cells in the draining lymph nodes to initiate adaptive immune responses. These antigen-presenting cells (APCs) include large numbers of Langerhans cells (LCs), dermal dendritic cells (DCs), macrophages and monocytes as well as accessory cells such as keratinocytes [6], [7], [8], [9]. As a result, vaccines administered to the skin have been shown to require a lower dose and/or generate stronger immune responses compared to conventional intramuscular or subcutaneous injection [5], [10].

Although vaccine delivery to the skin is attractive, vaccines are not usually administered this way in large part because convenient and reliable methods do not currently exist. Intradermal injection requires specialized training and is often unreliable [11]. Scarification also requires training and delivers an inefficient and variable dose [2], [3]. Other methods are under investigation, including small hollow needles [12], [13] scraping the skin with sandpaper or other structures [14], [15] and application of electrical or ultrasonic forces [10], [16].

In this study, we examine the use of vaccine-coated microneedles to deliver an inactivated H3N2 influenza vaccine into the skin. These micron-scale needles can be fabricated using low-cost methods for inexpensive mass production; prepared in a patch-like format for simple administration, possibly by patients themselves; and inserted painlessly across the stratum corneum and into the skin's epidermis and dermis to access the resident dendritic cells [17]. The use of microneedles has the potential of both increasing the immunologic response by targeting the skin and providing a simple-to-use delivery system for reliable administration without specialized training.

In this approach, a microneedle array is pressed manually against the skin and left in place for a few minutes while the vaccine coating dissolves off within the skin, after which the microneedles are discarded. In this study, the effectiveness of this technology was tested in mice after a single immunization of inactivated H3N2 virus and lethal challenge with a homologous mouse adapted strain. H3N2 strains are the leading cause of mortality and hospitalization rates in influenza-associated epidemics [18], [19] and improvements in vaccination would therefore have great health benefits. We hypothesize that microneedle-based vaccination will provide protective immunity against influenza with at least equivalent efficacy and improved applicability relative to current vaccination methods.

## Materials and Methods

### Microneedle fabrication

Microneedles were fabricated from stainless steel sheets (Trinity Brand Industries, SS 304, 75 µm thick; McMaster-Carr, Atlanta, GA) by laser cutting as described before [20]. To deburr and clean the microneedle edges and to make the tips sharp, microneedles were electropolished (model no. E399-100, ESMA, IL) in a solution containing glycerin, ortho-phosphoric acid (85%) and deionized water in a ratio of 6∶3∶1 by volume (Fisher Scientific, Fair Lawn, NJ). The final microneedle geometry was a linear array of five needles with a needle-to-needle spacing of 1575 µm. Each needle was 700 µm long, measured 170 µm by 55 µm in cross section at the base and tapered to a tip with a 5 µm radius of curvature.

### Micro-dip-coating

Microneedles were coated using a dip-coating process with a specially formulated coating solution described before [21], [22] containing 1% (w/v) carboxymethylcellulose sodium salt (low viscosity, USP grade, Carbo-Mer, San Diego, CA), 0.5% (w/v) Lutrol F-68 NF (BASF, Mt. Olive, NJ), 15% (w/v) D-(+)-trehalose dihydrate (Sigma, St. Louis, MO) and 5 mg/ml inactivated A/Aichi/2/68 (Aichi) virus. In order to reach high coating efficiency, the virus was concentrated by microfiltration using 300 kDa cut-off filters (Vivaspin 500, Sartorius Stedim Biotech, Germany).

**Figure 1:**
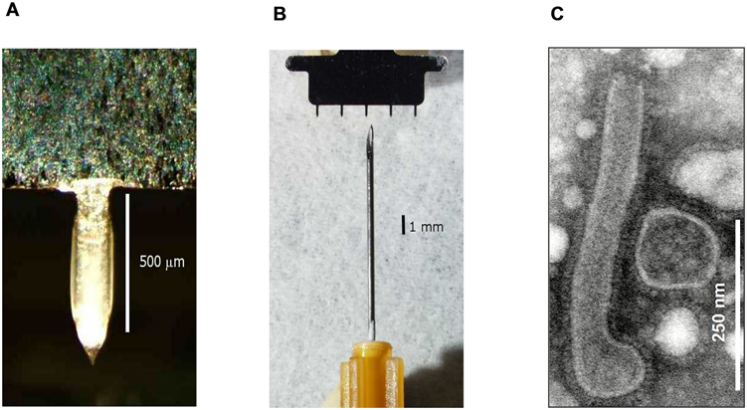
Photograph of (A) a single microneedle coated with influenza vaccine and (B) a five-needle array of microneedles shown with a 26 gauge hypodermic needle. (C) Electron microscopy of Aichi virus, showing a mixture of filamentous and spherical virus particles. The length of filamentous forms was between 450–500 nm.

The coating was performed manually by dip coating using an apparatus described elsewhere [21], and monitored by a video camera (DP71, Olympus America, Center Valley, PA) attached to a microscope (SZX16, Olympus America) (Figure 1A, 1B).

To measure the amount of vaccine coated per row of microneedles, three rows from each batch of coated microneedles were submerged into 200 µl of PBS buffer per row for 5 min. Vaccine dose was determined by measuring the concentration of protein in the solution as measured by BCA protein assay (Pierce Biotechnology, ThermoFisher Scientific, Rockford, IL) coupled with a calibration curve generated using known concentrations of Aichi virus.

### Cells and virus stocks

Madin-Darby canine kidney (MDCK) cells (ATCC CCL 34, American Type Culture Collection, Manassas, VA) were maintained in Dulbecco's Modified Eagle's Medium (DMEM) (Mediatech, Herndon, VA) containing 10% fetal bovine serum (Hyclone, ThermoFisher Scientific, Rockford, IL). Influenza virus stocks (Aichi) were prepared and purified as described [23]. The purity of the virus was determined by SDS PAGE followed by Coomassie blue staining and electron microscopy [23]. The hemagglutination (HA) activity was determined using bovine red blood cells (LAMPIRE Biological Laboratories, Pipersville, PA) as previously described [24]. For inactivation, the purified virus was treated with formalin at a final concentration of 0.1% (vol/vol), incubated for 72 h at 4°C, and then dialyzed against PBS buffer. Inactivation of virus was confirmed by plaque assay in MDCK cells [25].

The mouse-adapted Aichi strain was obtained by passage 8 times in Balb/c mice. The LD_50_ was determined in the same mouse strain and the viral titer was determined as mentioned above.

### Electron microscopy

To confirm the quality and purity of the virus preparation, purified virus was applied to a formvar carbon coated grid for 1 min, and immediately stained with 1% uranyl acetate for 30 s [26]. Excess stain was removed by filter paper, and the samples were examined by transmission electron microscopy (Figure 1C).

### Immunizations

Female Balb/c mice (Charles River Laboratory, Wilmington, MA) (11 mice per group, 6–8 weeks old) were immunized once by microneedle array (MN) or intramuscular (IM) immunization. For MN delivery, the mice were anesthetized with a ketamine and xylazine cocktail and the dorsal caudal surface was prepared to remove hair as described [23]. MN were then manually inserted into the skin, left in place for 2 min, and then removed. Immunization with approximately 3 µg or 10 µg of vaccine was accomplished by inserting one or three arrays, respectively, of 5 MN, each coated with 2.8±0.4 µg of vaccine. IM immunization was carried out by injecting either 3 µg or 10 µg vaccine suspended in 50 µl of PBS into the upper quadrant of the gluteal muscle.

### Challenge of mice with influenza virus

To determine post-challenge survival rates and immune responses, 6 mice per group were challenged 2 months after their immunization by intranasal instillation of 50 µl of virus with 5×LD_50_ (180 p.f.u.) of live mouse adapted Aichi virus and monitored for 14 days. As a negative control group, we included 6 naïve mice. A weight loss exceeding 25% in all infected mice was used as the experimental end-point, and mice reaching this end-point were euthanized according to IACUC guidelines. The challenged mice were monitored daily for signs of morbidity (body weight changes, fever and hunched posture) and mortality.

### Measurement of lung viral titers after challenge

To determine the titers of influenza virus in the lungs of immunized mice after challenge, the remaining five animals of each group were infected with 5×LD_50_ and were sacrificed on day 4. Naïve mice and naïve mice infected with 5×LD_50_ were used as control groups. Lung homogenates were prepared in DMEM serum-free medium to assess the viral titers per gram of tissue by plaque assay as described above.

### Sample collection

The animals were bled on days 14 and 28 after immunization and on day 4 after challenge. Blood was collected from the retro-orbital plexus with non-heparinized microcapillaries (Fisher Scientific, Pittsburgh, PA) after systemic anesthesia. Lung suspensions were prepared by mincing through a cell strainer (BD Falcon, Chelmsford, MA) and the cells were resuspended in DMEM. Lung cells were used in enzyme-linked immunospot (ELISPOT) assays whereas lung supernatants were mixed with the protease inhibitor phenylmethylsulfonyl fluoride (1 mM) (Sigma, St. Louis, MO) and stored at −20°C until assayed for viral titers and antibody-secreting cells. Inguinal lymph nodes and spleens were processed similarly into single cell suspensions in complete RPMI 1640 for cytokine determination [26]. All tissue samples were treated with red blood cell lysis buffer after their initial processing (Sigma, St. Louis, MO).

### Evaluation of humoral immune responses

All sera and lung supernatants were individually collected, and anti-Aichi specific IgG, IgG1, IgG2a and IgA antibody levels were determined quantitatively by enzyme-linked immunosorbent assay (ELISA) as described [26]. Purified mouse IgG, IgG1, IgG2a, IgA and goat anti-mouse-HRP for ELISA were purchased from Southern Biotechnology Associates (Birmingham, AL). Optical density was read at 450 nm.

We determined the hemagglutination inhibition (HAI) titers based on the WHO protocol [27] as described previously [23]. Heat-inactivated sera were treated with receptor-destroying neuraminidase (RDE) (Roche Diagnostics, Indianapolis, IN) overnight at 37°C, heat inactivated for 30 min at 56°C and incubated with packed chicken RBC for 1 h at 4°C to remove any cryoglobulins interfering with the agglutination reaction. They were serially diluted and pre-incubated at room temperature with 4 HA Units/50 µl of Aichi virus for 30 min. An equal volume of 0.5% chicken red blood cells was then added to each well for 30 min incubation at room temperature. The HAI titer was read as the reciprocal of the highest dilution of serum that conferred inhibition of hemagglutination. The values were expressed as the geometric mean+/−standard error of the mean.

### Evaluation of systemic cellular immune responses

Spleen and lymph node cells were prepared from mice euthanized 4 days after challenge with 5×LD_50_ (180 p.f.u.) mouse adapted virus. Cell suspensions (0.5–1×10^6^/well) were stimulated *in vitro* in the presence of 1 µg/ml nucleoprotein (NP) peptide stimulants or Aichi virus in complete RPMI medium (cRPMI) [28]. As NP immunogen we used the H-2^d^-restricted NP class I peptide (TYQRTRALV) and a pool of three H-2^d^-restricted class II peptides (FWRGENGRKTRSAYERMCNILKGK, RLIQNSLTIERMVLSAFDERNK, and AVKGVGTMVMELIRMIKRGINDRN) [29], [30]. Culture supernatants were collected at 12 h, 48 h and 72 h and stored at −80°C until assayed for IFN-γ, IL-4 and IL-2 levels, according to the manufacturer's instructions (eBioscience, San Diego, CA).

### Quantification of anti-Aichi antibody-forming cells and memory B cells

Virus-specific antibody-secreting cells (ASC) in the spleen and the lungs were determined by a modification of the ELISPOT assay [31], [32], [33]. Briefly, 96-well plates were coated overnight at 4°C with purified inactivated Aichi virus at a final concentration 4 µg/ml. The plates were washed three times with RPMI and blocked for 2 h with 10% fetal calf serum prior to sample addition. Splenocyte and lung single cell suspensions collected at 4 days after challenge (0.5–1×10^6^/well) in cRPMI were plated directly on coated blocked plates and were incubated at 37°C for 36 h. Virus-specific ASC were detected as spots after incubation with goat anti-mouse IgG horseradish peroxidase (Southern Biotechnology Associates) and developed with stable diaminobenzidine (Research Genetics). Samples were enumerated in an ELISPOT reader (Cellular Technology, Shaker Heights, OH) and the results shown as the number of ASC per 10^6^ cells.

The ELISPOT assay to detect memory B cells was carried out similarly, except that splenocytes were stimulated to induce differentiation of memory B cells into ASC. Cell suspensions (5×10^5^/well) were incubated for 1 and 6 days at 37°C in cRPMI supplemented with 10% Concavalin A and lipopolysaccharide-conditioned medium (ConA media) [34] with or without 1 µg/ml Aichi virus. At the end of the incubation, the cells were washed twice with complete medium and transferred into virus-coated plates as described above. To estimate the number of influenza-specific memory B cells, the number obtained from unstimulated samples (negative control) was subtracted from those in the influenza-stimulated samples. ASC numbers of vaccinated mice on day 1 or day 6 were considered positive if the numbers of spots were higher than the sum of naïve infected group ASC numbers plus 3 times the standard deviation [35].

### Statistics

The statistical significance of the difference was calculated by two-tailed unpaired Student's t-test and one-way ANOVA (one–way analysis of variance including Bonferronis's multiple comparison test). Values were considered significant for p≤0.05. Unless otherwise stated, experiments were run at least in duplicates.

## Results

### Induction of hemagglutination inhibition and antibody responses after microneedle immunization with inactivated influenza virus

**Figure 2:**
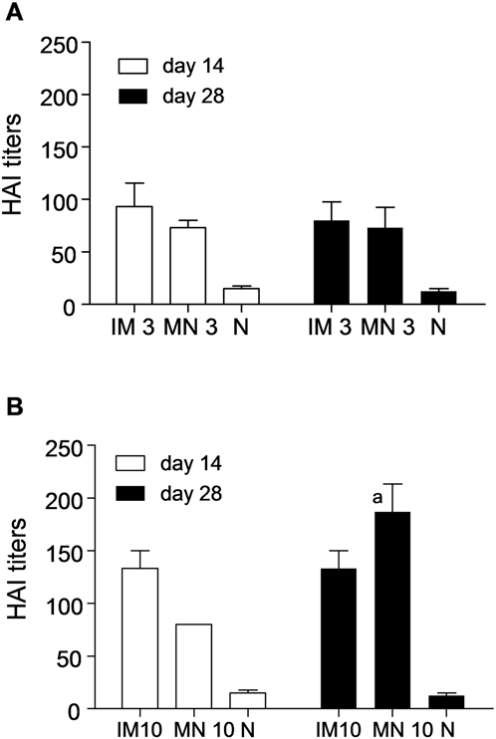
Influenza-specific hemagglutination inhibition (HAI) titers. HAI titers of sera from mice immunized with (A) 3 µg and (B) 10 µg inactivated influenza virus administered by intramuscular (IM) and microneedle (MN) routes of delivery compared to naïve (N) mice at 14 and 28 days after a single immunization (graphs express average±s.e) ^a^p<0.05 when MN 10 µg is compared to the IM 10 µg group.

To evaluate the efficacy of transdermal immunization with whole inactivated A/Aichi/2/68 (Aichi) H3N2 influenza virus, we determined the hemagglutination inhibition (HAI) titers as correlates of immunity in the sera of immunized mice. Groups of mice were immunized on the caudal site of the dorsal skin using solid microneedle arrays (MN) coated with 3 or 10 µg of inactivated virus (Figure 1). In parallel we immunized mice intramuscularly (IM) with the same doses of the virus to be used for comparison. We bled the animals at 14 days and 28 days after a single immunization and assessed HAI titers in the sera. After immunization at the lower dose (3 µg), we observed a marked increase in the HAI titers of the MN group, close to 80, which remained elevated at 28 days (Figure 2A). Comparable responses were observed with IM immunization using the same virus dose (Figure 2A). When a higher antigen dose (10 µg) was used, the HAI titers increased relative to the lower dose, but were only statistically higher for the MN group at day 28 (titer: 187, p = 0.0035)(Figure 2B). Moreover, titers in the MN group were significantly higher than the IM group at the 10 µg dose at day 28. These results demonstrate that a single MN immunization with inactivated influenza virus induces high titers of functional antibodies at levels at least comparable to those observed by IM injection.

**Figure 3:**
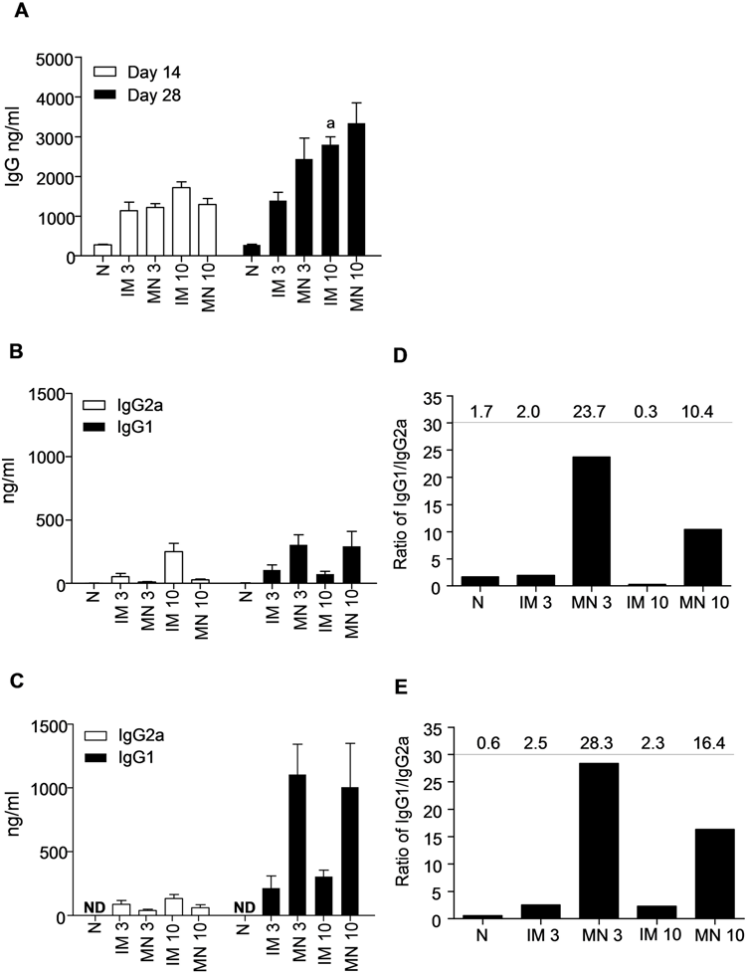
Total anti-influenza serum IgG and isotype profiles after intramuscular (IM) and microneedle (MN) immunization with 3 µg or 10 µg of inactivated influenza virus compared to naïve control group (N). (A) Total serum IgG titers determined on days 14 and 28 after immunization. (B) Serum IgG2a and IgG1 isotypes on day 14 and (C) Serum IgG2a and IgG1 isotypes on day 28. (D) Ratio of IgG1/IgG2a on day 14 (E) Ratio of IgG1/IgG2a on day 28. All IgG measurements were made by quantitative ELISA (average±s.e) ^a^p<0.05 when IM 10 µg compared to the IM 3 µg group.

We also measured the circulating levels of influenza-specific IgG in sera by ELISA (Fig. 3). We observed a substantial increase in influenza virus-specific IgG levels 14 days after immunization in the sera of immunized mice as compared to the naïve group (p<0.001) (Figure 3A). By day 28, the anti-Aichi antibody titers further increased twofold in the immunized mice with the exception of the 3 µg IM immunized group. We only observed a dose-dependent increase in response for the IM group. The high IgG titers obtained by MN delivery at both doses suggest that it performs better than IM injection at low doses.

Finally, we examined if the route of immunization influenced the isotype profile. We found that the IM-immunized groups on day 14 after immunization exhibited IgG1/IgG2a ratios of 2 and 0.3 with 3 µg and 10 µg inactivated virus, respectively (Figure 3B, D). In contrast, the MN-immunized groups had IgG1/IgG2a ratios of 24 and 4 in the 3 µg and 10 µg groups, respectively. By day 28, there were small increases in the IgG1/IgG2a ratios in all groups, but the trends were unchanged (Figure 3C, D). This is significant because the ratio of IgG1/IgG2a is correlated with immune responses that are predominantly T helper type 2 (Th2) or T helper type 1 (Th1), suggesting a strong bias towards Th2 responses with the MN route and Th1 with the IM route. This observation indicates a significant difference in the nature of the immune response generated using MN delivery to the skin as opposed to IM injection.

### Body weight changes, survival rates and effective clearance of virus from lungs of challenged mice

**Figure 4:**
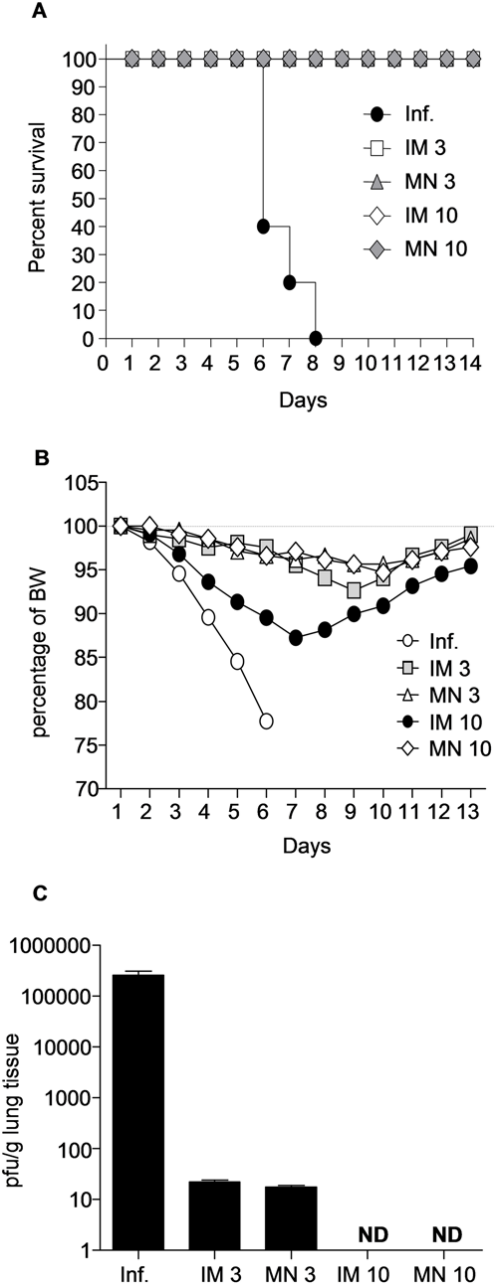
Protective efficacy from influenza virus challenge. Post-challenge survival rates of immunized and control mice were monitored for 14 days. (A) Percent survival after challenge with 5×LD_50_ with mouse adapted Aichi virus of intramuscularly (IM) or microneedle array (MN) immunized mice (B) Normalized body weights (BW) of surviving mice recorded during the infection period. (C) Lung viral titers were assessed as an indicator of protection after intranasal challenge with 5×LD_50_ of live Aichi virus, (average±s.e). Lungs were harvested on day 4 and lung extracts were used to determine viral titers by plaque assay using MDCK cells. (Inf.) unimmunized infected mice ND: not detected.

The efficacy of a successful vaccine is shown by the protection induced against lethal challenge with the homologous virus. Mice from both IM and MN groups were challenged with 5×LD_50_ of homologous, highly pathogenic mouse-adapted virus 2 months after immunization. All vaccinated mice survived challenge and lost less than 10% of their original body weight (Figure 4A, B), while the naïve counterparts died within 8 days after challenge.

To further evaluate the protective response, we also examined the extent to which MN-induced immune responses controlled viral replication in the lungs *in vivo*. The unimmunized infected group displayed very high viral titers by day 4 post-infection. In contrast, groups of immunized mice showed at least a 5-log_10_ decrease in viral titers when compared to the naïve group (Figure 4C). Notably, we could not detect any virus in the lungs of either the MN or IM group at 10 µg. The 3 µg MN and IM groups had minimal levels of virus on day 4 (4-log_10_ lower than the naïve group) showing that even at this lower vaccination dose the mice effectively cleared almost all the virus. These data indicate that MN immunization with inactivated influenza virus can successfully prevent viral replication and confer protection against lethal viral challenge similarly to IM injection.

### Recall humoral immune responses in challenged mice

**Figure 5:**
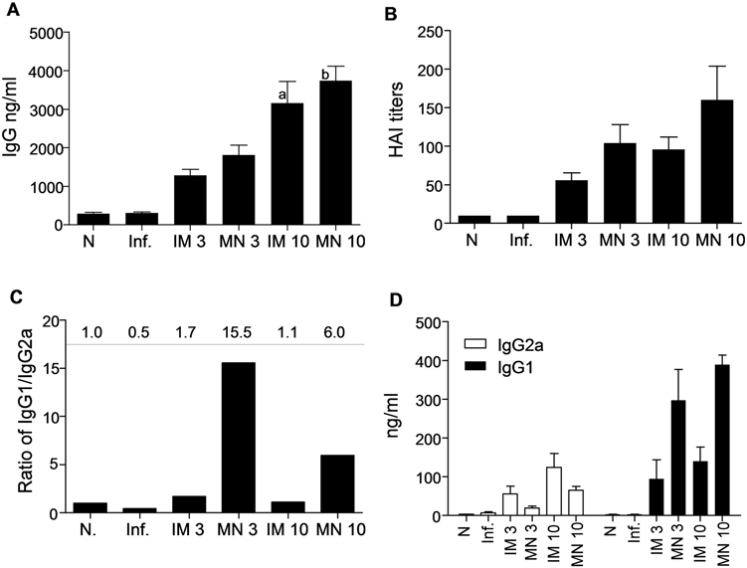
Humoral immune responses 4 days after challenge. (A) Total serum IgG titers determined with quantitative ELISA and (B) (B) HAI titers of sera from immunized mice. (C,D) IgG2a and IgG1 isotype titers were determined with quantitative ELISA (average±s.e). (N) Naïve control, (Inf.) unimmunized infected mice ^a^p<0.05 when IM 10 µg is compared to the IM 3 µg group. ^b^p<0.05 when MN 10 µg is compared to the MN 3 µg group.

Because induction of neutralizing antibodies is a significant factor in protection from homologous viral challenge, we compared the humoral immune responses responsible for the rapid virus clearance. Immunized groups of mice challenged with 5×LD_50_ of mouse adapted Aichi virus 2 months after immunization demonstrated robust humoral responses that increased with dose (p = 0.015 when comparing 10 µg IM vs 3 µg IM, and p = 0.0017 for 10 µg MN vs 3 µg MN respectively), but showed no significant differences between the IM or MN immunizations (Figure 5A). The post-challenge HAI titers demonstrated similar dose-dependent responses in both routes of immunization (Figure 5B). The isotype profiles were similar to the pre-challenge data, maintaining the observed skewed responses toward Th2 for the MN immunization and Th1 for the IM immunization (Figure 5C, D).

### *In vitro* cytokine responses

**Figure 6:**
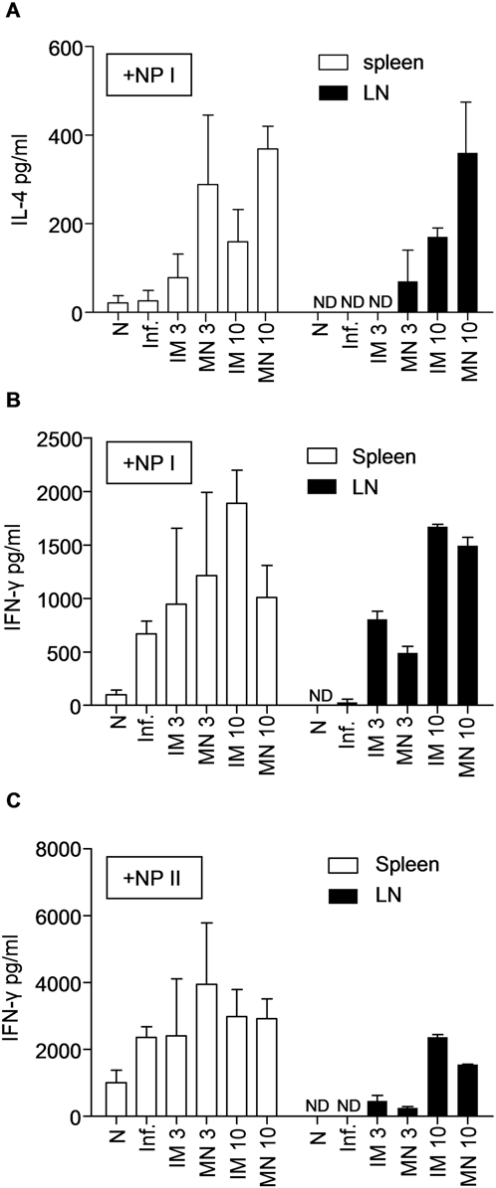
IL-4 and IFN-γ secretion by T cells in response to inactivated influenza antigen. The frequency of cellular immune responses is shown in immunized and challenged mice. Spleens and lymph nodes from all groups of immunized mice were individually processed and cells were cultured in the presence of NP Class I or NP Class II peptides for stimulation. (A) Splenocytes and lymphocytes were stimulated with NP Class I peptide and analyzed with ELISA for IL-4 production at 72 hours. Splenocytes and lymphocytes cultured in the presence of NP Class I (B) or NP Class II stimulants (C) were analyzed for IFN-γ production at 72 hours after stimulation. (N) Naïve control, (Inf.) unimmunized infected mice (average±s.e). ND: not detected. ^a^p<0.05 when MN 10 µg compared to the IM 10 µg group. ^b^p<0.05 when IM 10 µg compared to the MN 10 µg group.

To assess cellular immune responses, we re-stimulated the splenocytes and lymph node cultures (LN) isolated from challenged mice on day 4 with NP Class I and II restricted peptides, and quantified their ability to produce cytokines (IL-2, IL-4, IFN-γ) by ELISA (Figure 6). The production of IL-2 did not show significant differences in splenocyte or LN cultures re-stimulated with Aichi virus or NP Class I and II peptides (data not shown).

In both splenocytes and LN cultures the IL-4 levels did not show any significant differences between immunized or unimmunized infected groups after NP Class II re-stimulation (data not shown). In contrast, after NP Class I re-stimulation, both cell populations showed a dose-dependent increase in IL-4 production. LN IL-4 responses increased significantly for both the MN (p = 0.06) and the IM groups (p = 0.0005). Particularly, the 10 µg MN group produced a two fold higher level of IL-4 after re-stimulation when compared to the 10 µg IM group (p = 0.0189) (Figure 6A). Elevated IL-4 production after MN vaccination is consistent with the heightened Th2 driven responses as indicated by the elevated IgG1/IgG2a ratio observed in the MN groups, since IL-4 production is associated with class switching [36].

IFN-γ levels did not significantly change in splenocyte cultures of any groups after NP Class I or NP Class II re-stimulation (Figure 6B, C). However, LN cells re-stimulated with NP Class I peptides produced a dose-dependent IFN-γ secretion that was independent of the route of vaccination (Figure 6B). NP Class II peptide-stimulated LN cultures showed a greater increase in IFN-γ response in the IM immunized group compared to the MN counterpart at the high antigen dose (p<0.05). Elevated IFN-γ production after IM vaccination is consistent with the heightened Th1-like response indicated by the reduced IgG1/IgG2a ratio in the IM groups, since IFN-γ production is associated with IgG2a production [37].

### Virus-specific antibody secreting cells in immunized mice

**Figure 7:**
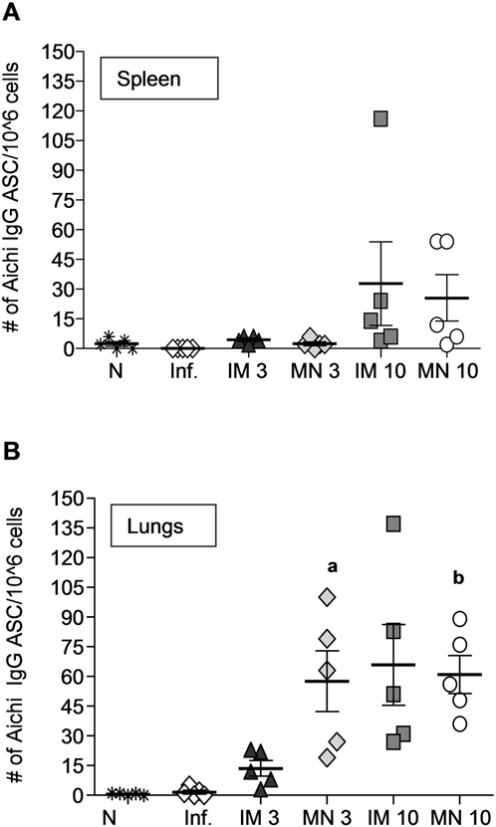
Antibody secreting cells (ASC) in spleens and lungs of immunized mice. (A) Splenocytes and (B) single cell suspensions from lungs of immunized mice were assessed by ELISPOT assay for anti-influenza IgG ASC and compared with (N) naïve control, and (Inf.) unimmunized infected groups 4 days post–challenge (average±s.e). ^a^p<0.05 when MN 3 µg compared to the IM 3 µg group. ^b^p<0.05 when MN 10 µg compared to the IM 3 µg group.

The sustained humoral immune responses that confer protection against influenza infection are attributed in part to long-lived antibody-secreting cells (ASC). Eight weeks after the immunization and shortly after challenge, the mice were examined for ASC in spleen and lungs. In spleen, anti-Aichi IgG ASC were only elevated in MN and IM groups which received the 10 µg dose, with no significant differences among these groups. Antigen-secreting cell numbers were very low in all other groups including immunized, naïve or infected mice (Figure 7A).

In contrast, virus-specific IgG ASC in the lungs were elevated in the MN groups at both antigen doses and in the IM group at high antigen dose, reflective of the site of infection. Importantly, the MN 3 µg group had 4-fold higher numbers than the IM group at the same dose (p = 0.05) (Figure 7B). These data demonstrate the generation of local humoral responses by MN vaccination.

### Induction of memory B cell responses by microneedle immunization

In contrast to the non-dividing, terminally differentiated ASC, which spontaneously secrete large antibody amounts without any antigenic stimulation *ex vivo*, memory B cells need antigenic stimulation to divide and differentiate into ASC [35]. Memory B cells are highly specialized cells that are induced by minimal amounts of antigen to become antibody-secreting cells conferring long-term protection [38] and hence important to vaccine development. To determine whether MN vaccination could result in the induction of virus-specific B cell memory, splenocytes were isolated from vaccinated or naïve mice after challenge with live mouse-adapted virus. The cells were stimulated with Aichi virus and used to identify Aichi-specific ASC by ELISPOT assay.

**Figure 8:**
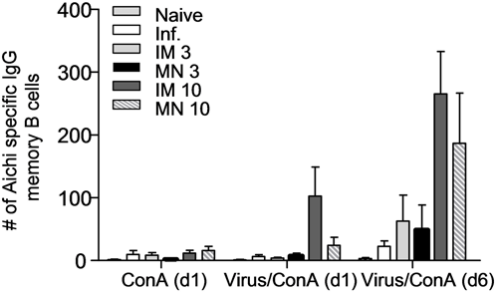
Induction of anti-influenza-specific IgG memory B cells in spleens of immunized mice. Splenocytes were stimulated with inactivated whole virus for 6 days in the presence of ConA medium to assess the generation of anti-influenza IgG memory B cell by ELISPOT, (average±s.e). ConA (d1): ASC cells stimulated with ConA medium and enumerated on day 1.Virus+ConA (d1): memory B cells stimulated with influenza virus as an antigen and ConA medium and enumerated on day 1. Virus+ConA (d6): memory B cells stimulated with influenza virus and ConA medium and enumerated on day 6.

We observed a significant increase in memory B cells over a period of 6 days (Fig. 8), whereas these numbers were very low in the control groups supplemented with Con-A conditioned medium alone (Figure 8). On day 6, all groups presented increased ASC numbers by 2 to 10 fold when compared to day 1, including the unimmunized infected mice. We detected a 2-fold higher increase in the ASC numbers of mice immunized with 3 µg of virus as compared to the naïve infected mice and marked 2- to 3-fold increases in the 10 µg IM and MN groups. These data indicate that increased vaccine doses correlated with increased numbers of memory B cells. The similarity in memory B cell numbers observed after IM and MN immunization demonstrates that MN immunization was as effective as IM in inducing memory B cell responses.

## Discussion

This study was motivated by the need to improve immunization against influenza specifically and other diseases in general. Our main goal was to determine if MN delivery of influenza vaccine could induce comparable immune responses to the conventional IM injection, using a MN array with numerous logistical advantages. We conclude that MN immunization can indeed provide protective immunity at least as strong as that of IM injection, with the notable difference that MN vaccination shifted the immune response significantly toward a Th2 bias.

More specifically, this study showed that vaccination by the new technology of vaccine-coated MN arrays could induce strong humoral and cellular immune responses that can effectively confer full protection with a single immunization. Solid metal MN coated with inactivated influenza virus conferred 100% protection against lethal viral challenge and induced short-lived antigen-secreting cells in the lungs and spleens of immunized mice as well as anti-influenza memory B cell responses. MN immunization was found to be at least as effective as the conventional IM route in eliciting similar levels of functional antibodies at low or high antigen concentrations, in clearing the virus from the lungs of infected mice, in conferring protection and in inducing short-lived as well as memory B immune responses as seen by the frequency of antigen-secreting cells in spleens of challenged mice. Other approaches for transdermal and intradermal immunization have also been recently reported [4], [12], [13], [39], [40], [41].

Interestingly, we observed differences in the responses between IM and MN immunization that may have implications for vaccine development. In IM immunization, the serum IgG responses were dose related and the Th1 responses were predominant as shown by the isotype profile before and post-challenge and the cytokine data. In contrast, responses to MN immunization were similar to each other at both antigen concentrations and both doses were equally potent in inducing similar influenza-specific IgG titers. In addition the transdermal delivery with MN induced robust Th2 responses as seen by the IgG1/IgG2a ratios.

Delivery of antigen into the skin by MN, which are inserted into the epidermis and the upper layer of the dermis, may activate both LC and dermal dendritic cells (DCs) which in turn convey the antigen to the draining lymph nodes [1]. Recent studies have demonstrated the tissue prevalence of several DC subsets and their fundamental role in controlling the adaptive immune response [42], [43]. In this study, we observed that MN immunization successfully elicited Th1 and Th2 responses with a single dose. Interestingly, the cellular and humoral responses generated using the transdermal route were skewed predominantly to Th2 when compared to IM, likely a consequence of higher levels of IL-4 secretion from spleen and inguinal lymph nodes. Although IL-4 secretion was seen by both CD4+ and CD8+ T cell lymphocytes, the differences in IL-4 levels were mainly attributed to the CD8+ population as seen by the NP Class I stimulation of lymphocytes, because neither polyclonal stimulation with inactivated Aichi virus nor the presence of NP Class II peptides revealed any distinct differences among immunized groups. The levels of CD4+ associated IFN-γ were similar in both spleens and lymph nodes of IM and MN groups whereas the CD8+ associated IFN-γ was higher in the lymphocytes of the IM immunized animals. The generation of a broad spectrum of T cell responses and the differences observed by the administration route deserve further consideration since recent reports have demonstrated the importance of T cell memory responses in heterologous protection, especially in influenza cases of antibody-escape variants [44], [45].

It has been reported that elderly people, who face higher risk from influenza infection, have decreased IL-2 production, diminished cytotoxic T-lymphocyte (CTL) activity [46], [47], [48] and changes in T helper cell memory affecting the Th1 responses to influenza infection [49]. Achieving the most effective immune response against influenza may require an optimal balance of Th1 and Th2 responses. The development of a strategy that incorporates the use of adjuvants in MN delivery may enable this balance to be achieved.

The induction of ASC in the lungs, which are the site of virus entry and replication, is important to maintain sustained immune responses after vaccination. We found that both concentrations of antigen delivered with MN induced the same numbers of ASC in the lung, whereas the 3 µg IM induced lower levels of these cells. Although we did not observe any significant IgA levels in the mucosa (data not shown), It is likely that one immunization is not sufficient to induce high IgA titers.

In addition to the immunologic similarities and differences between MN and IM delivery, the use of MN presents several logistic advantages for vaccination. First, MN inherently delivers antigen to the APC in the skin by targeting mainly the epidermis and the upper layers of dermis. This approach may provide dose sparing as suggested by data shown in this study as well as previous studies [50], [51]. Influenza vaccine production capacity has limited access to seasonal vaccine in some recent years and, in the event of a future influenza pandemic, vaccine supply will almost certainly be insufficient [52]. Thus, dose-sparing approaches are important to ensuring an adequate supply of influenza vaccine.

Another advantage of MN-based delivery is the simplicity of MN administration compared to hypodermic injection. MN arrays can be integrated into patches that can be applied by manual pressing against the skin. Such MN patches are envisioned to require no special training and can be suitable for administration by minimally trained personnel or by patients themselves. Microneedles have also been shown to be painless in contrast to hypodermic needles [53]. These features of microneedles could increase coverage of seasonal and pandemic influenza vaccination by facilitating school-based vaccination of children, self-administration by busy adults, and easy access to vaccination in elder-care facilities or at home, which would minimize risks of long delays associated with injection-based vaccination at centralized clinics. This contrasts with the specialized training needed for IM hypodermic injection and the even more specialized training, and noted unreliability, of intradermal hypodermic injection [11].

Other advantages include the small package size of MN, which reduces storage, transportation and disposal volumes and associated costs. A MN patch is small enough to fit inside an envelope for delivery by the postal service and will occupy much less space in the cold chain, of critical importance in developing countries. MN should reduce the dangers of accidental and intentional hypodermic needle re-use and thereby increase safety. Coating MN with a dry vaccine formulation that inherently avoids the need for reconstitution should prolong their shelf life and resistance to elevated temperatures. When mass-produced, MN are expected to have a cost similar to that of a conventional hypodermic needle and syringe [17]. Solid metal MN delivery offers the potential to increase the vaccine coverage not only for prevention of influenza, but also for other infectious diseases.

Future MN immunization studies are warranted in other animal models including guinea pigs, pigs or ferrets, whose skin thickness is closer to that of human skin. Additional dose-response studies are needed to define the minimum antigen dose that will confer full protection against lethal viral infection. Challenge studies with higher LD_50_ levels will also provide further insight into the potency of MN immunization to induce protection. Another very important topic for future study is to determine the potential of MN immunization to enhance the breadth of immunity against homologous and heterologous influenza strains.
